# Maternal Particulate Matter Exposure Impairs Lung Health and Is Associated with Mitochondrial Damage

**DOI:** 10.3390/antiox10071029

**Published:** 2021-06-25

**Authors:** Baoming Wang, Yik-Lung Chan, Gerard Li, Kin Fai Ho, Ayad G. Anwer, Bradford J. Smith, Hai Guo, Bin Jalaludin, Cristan Herbert, Paul S. Thomas, Jiayan Liao, David G. Chapman, Paul S. Foster, Sonia Saad, Hui Chen, Brian G. Oliver

**Affiliations:** 1 Faculty of Science, School of Life Sciences, University of Technology Sydney, Ultimo, NSW 2007, Australia; Baoming.Wang@student.uts.edu.au (B.W.); Yik.chan@uts.edu.au (Y.-L.C.); gerard.E.li@student.uts.edu.au (G.L.); David.Chapman@uts.edu.au (D.G.C.); hui.chen-1@uts.edu.au (H.C.); 2 Respiratory Cellular and Molecular Biology, Woolcock Institute of Medical Research, The University of Sydney, Sydney, NSW 2037, Australia; 3 Jockey Club School of Public Health and Primary Care, The Chinese University of Hong Kong, Hong Kong, China; kfho@cuhk.edu.hk; 4 ARC Centre of Excellence for Nanoscale Biophotonics, Faculty of Engineering, Graduate School of Biomedical Engineering, UNSW Sydney, Sydney, NSW 2052, Australia; a.anwer@unsw.edu.au; 5 Department of Bioengineering, Department of Paediatric Pulmonary and Sleep Medicine, School of Medicine, University of Colorado, Boulder, CO 80309, USA; Bradford.smith@cuanschutz.edu; 6 Air Quality Studies, Department of Civil and Environmental Engineering, Hong Kong Polytechnic University, Hong Kong, China; ceguohai@polyu.edu.hk; 7 Ingham Institute for Applied Medical Research, University of New South Wales, Sydney, NSW 2052, Australia; b.jalaludin@unsw.edu.au; 8 Centre for Air Pollution, Energy and Health Research (CAR), Woolcock Institute of Medical Research, The University of Sydney, Sydney, NSW 2037, Australia; 9 Department of Pathology, Faculty of Medicine, School of Medical Sciences, Prince of Wales’ Clinical School, University of New South Wales, Sydney, NSW 2052, Australia; c.herbert@unsw.edu.au (C.H.); paul.thomas@unsw.edu.au (P.S.T.); 10 Institute for Biomedical Materials and Devices, Faculty of Science, University of Technology Sydney, Ultimo, NSW 2007, Australia; Jiayan.Liao@student.uts.edu.au; 11 Priority Research Centre for Healthy Lungs, University of Newcastle, Callaghan, NSW 2308, Australia; Paul.Foster@newcastle.edu.au; 12 Renal Group, Kolling Institute of Medical Research, The University of Sydney, St Leonards, Sydney, NSW 2064, Australia; Sonia.saad@sydney.edu.au

**Keywords:** air pollution, lung function, reactive oxygen species, mitochondrial dysfunction, asthma

## Abstract

Relatively little is known about the transgenerational effects of chronic maternal exposure to low-level traffic-related air pollution (TRAP) on the offspring lung health, nor are the effects of removing such exposure before pregnancy. Female BALB/c mice were exposed to PM_2.5_ (PM_2.5,_ 5 µg/day) for 6 weeks before mating and during gestation and lactation; in a subgroup, PM was removed when mating started to model mothers moving to cleaner areas during pregnancy to protect their unborn child (Pre-exposure). Lung pathology was characterised in both dams and offspring. A subcohort of female offspring was also exposed to ovalbumin to model allergic airways disease. PM_2.5_ and Pre-exposure dams exhibited airways hyper-responsiveness (AHR) with mucus hypersecretion, increased mitochondrial reactive oxygen species (ROS) and mitochondrial dysfunction in the lungs. Female offspring from PM_2.5_ and Pre-exposure dams displayed AHR with increased lung inflammation and mitochondrial ROS production, while males only displayed increased lung inflammation. After the ovalbumin challenge, AHR was increased in female offspring from PM_2.5_ dams compared with those from control dams. Using an in vitro model, the mitochondria-targeted antioxidant MitoQ reversed mitochondrial dysfunction by PM stimulation, suggesting that the lung pathology in offspring is driven by dysfunctional mitochondria. In conclusion, chronic exposure to low doses of PM_2.5_ exerted transgenerational impairment on lung health.

## 1. Introduction

The World Health Organization reported that more than 91% of people are living in areas with air pollution [1]. Urbanisation has resulted in traffic-related air pollution (TRAP) being one of the most dominant air pollution sources. The complex composition of TRAP results in more severe impacts on human health than other airborne pollutants [2].

The pathological effects of TRAP are similar to cigarette smoking in that epidemiological studies have shown that exposure can both initiate respiratory diseases and cause symptoms in people with pre-existing lung disease such as asthma [3,4] and even affect the unborn child if exposure occurs during pregnancy [5,6]. The mechanisms by which this occurs are largely unknown. Inhaled particulate matter (PM) smaller than 2.5 µm in aerodynamic diameter (PM_2.5_) can reach the alveoli and enter the bloodstream [7]. Moreover, PM_2.5_ can remain airborne for long periods, which renders it the major toxic element in TRAP. TRAP PM contains several chemicals which are oxidants, such as copper, antimony and lead [8]. At the cellular level, oxidants can damage the mitochondria, resulting in the imbalance between the removal of damaged mitochondria and re-generation from the undamaged mitochondrial fragment. These processes are mediated through mitochondrial fission (separating damaged and healthy fragments) and fusion (combining healthy fragments), which is also called mitophagy that is essential to maintain mitochondrial homeostasis. Damaged mitochondria produce excessive reactive oxidative species (ROS), resulting in oxidative stress, inflammation and tissue damage [9,10]. Indeed, our previous studies have confirmed the close relationship between organ pathology and mitochondrial dysfunction [11].

Exposure to high levels of air pollution (e.g., as found in large cities in India and China) during pregnancy can decrease the placental growth factor [12], increase cord blood immune biomarkers (e.g., Ig E and IL-33) [13] and cause mitochondrial oxidative DNA damage [14]. Previous studies in mouse models using a high dose of PM have shown that in utero exposure to 100 μg PM collected from residential roof spaces impaired somatic growth and reduced lung volume and lung function in the offspring [15]. Pregnant mice exposed to combustion generated particles containing free radicals (200 nm, 50 μg) exhibited systemic oxidative stress and their offspring also showed an increased risk of developing asthma [16].

In the abovementioned studies, mice were exposed to relatively high levels of PM, which reflects the high levels of annual ambient air pollution in Asia and Africa [17]. However, the population-weighted mean annual PM concentrations in Europe, North America and Oceania are relatively low (5–15 µg/m^3^) and often considered as safe [17]. A recent study, including almost 1000 cities in Europe, demonstrated the positive association between premature mortality and air pollution when the concentration was below WHO recommendation [18]. In Australian cities, the most prominent areas with higher levels of PM exposure are close to major traffic corridors where population-dense residential high rise buildings are often located. Previously, we developed a novel mouse model of low dose TRAP PM exposure (replicating urban Sydney levels of 17 μg/m^3^) in which we found that exposure to 5 μg of TRAP PM_2.5_ for 3 weeks can elicit a significant inflammatory response in the lung [19]. Another study exposing mice to a single dose of mixed TRAP and other sources of PM at 5 µg or 15 µg PM also found lung inflammation and mechanical impedance in the lung [20]. The effects of low levels of air pollution are only just beginning to be appreciated and are often not understood by the general public and policymakers.

Therefore, this study aims to investigate whether maternal chronic exposure to low dose PM_2.5_ (5 μg/day) can affect lung health in both dams and offspring using a mouse model and whether these changes are related to dysfunctional mitochondria. We used a second insult in the offspring to investigate whether maternal PM exposure affects the development of allergic airway disease (i.e., asthma). Furthermore, some mothers may be conscious of their local environment and would move to areas with less traffic and better air quality at the onset of pregnancy. However, it is unknown if such an approach would protect their unborn child. Therefore, we modelled this situation in our study and referred to it as the Pre-exposure group in which PM exposure was only applied before pregnancy and removed at mating. This study provides much needed information on the risks of low-level PM exposure directly and prenatally, as well as whether simply removing the exposure during pregnancy is sufficient to benefit the lung health of offspring.

## 2. Materials and Methods

### 2.1. PM_2.5_ Preparation

PM_2.5_ were collected as previously described [19]. Briefly, in the summer (24 June to 11 July 2017), PM_2.5_ was collected in the busy roadside in Hong Kong by the URG PM samplers (URG-2000-30EH, 8 L/min) through a 47 mm Teflon (Pall Life Sciences, Ann Arbor, MI, USA) and (800 °C, 3 h) 47 mm quartz-fiber filters (Whatman, Clifton, NJ, USA). PM was extracted from the filters with 90% ethanol and 5 min of sonication and then it was dried overnight by a freeze dryer. The contents of organic carbon and elemental carbon were analysed using a Desert Research Institute Model 2001 Thermal/Optical Carbon Analyzer with the IMPROVE-A protocol. Water-soluble inorganic ions were determined by ion chromatography. PM size was determined by dynamic light scattering (Microtrac252, Montgomeryville, PA, USA). PM morphology was characterised by a transmission electron microscopy (TEM, JEOL TEM-1400, acceleration voltage 120 kV).

### 2.2. Animal Experiments 

**Figure 1:**
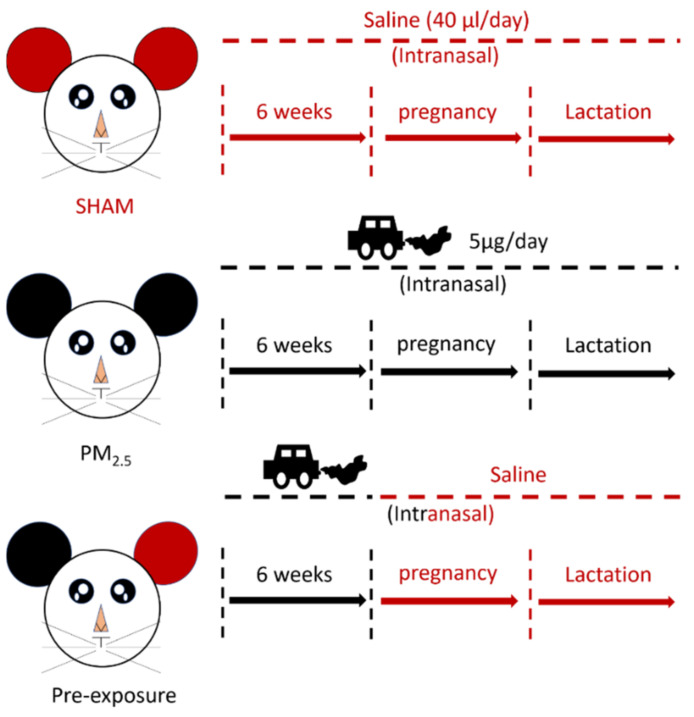
Flow chart of maternal exposure.

The animal experiments were approved by the Animal Care and Ethics Committee at the University of Technology Sydney (ETH17-1998), following the Australian National Health and Medical Research Council Guide for the Care and Use of Laboratory Animals. Virgin female BALB/c mice (6 weeks, Animal Resources Centre, Perth, WA, Australia) were divided into SHAM (saline, 0.9% sodium chloride), PM_2.5_ and Pre-exposure groups. The PM_2.5_ group was exposed to PM_2.5_ (5 μg/day intranasal installation) suspended in 40 μL saline (20 μL each naris) [19] prior to mating for 6 weeks during gestation and lactation, while the Pre-exposure group was exposed to the same amount of PM_2.5_ only during the six weeks pre-mating period to evaluate if this can protect lung health in both dams and offspring. The SHAM group was exposed to saline (40 μL in total, 20 μL each naris) for the same period (Figure 1). Since the prevalence of asthma is greater in adult females and in murine models, female mice have greater responses to ovalbumin (OVA) [21]; therefore, we only investigated allergic airways disease (asthma) in adult female mice.

### 2.3. Lung Function Tests

Lung function was measured by the forced oscillation technique (FlexiVent, SCIREQ, Montreal, QC, Canada). Dams and offspring were anesthetised (tribromoethanol, 250 mg/kg, Sigma-Aldrich, St Louis, MO, USA) and tracheostomised. Then, an 18 gauge polyethylene cannula was inserted. The cannula was connected to the FlexiVent and ventilated at 200 breaths/min with a tidal volume of 10 mL/kg and positive end-expiratory pressure of 3 cm H_2_O.

Lung function was performed at baseline and after increasing doses of methacholine (0, 1.6, 3.125, 6.25, 12.5, 25 and 50 mg/mL, Sigma-Aldrich, St Louis, MO, USA) generated by an in-line nebuliser in order to measure airway reactivity. Two deep inspirations were performed before each dose to standardise volume history. The impedance of the respiratory system was fit in order to calculate Newtonian resistance (Rn, reflecting airway resistance), tissue damping (G, reflecting tissue resistance) and tissue elastance (H, reflecting lung stiffness). The results were the mean of the three consecutive peak measurements at each dose.

### 2.4. OVA-Induced Airway Hyperresponsiveness

Female offspring (9 weeks) were sensitised with OVA (100 μg, ip.) on days 0 and 14, followed by aerosolised OVA (1%, 30 min) challenge on days 18, 21, 23, 25 and 27. The lungs were collected the next day after the last OVA administration.

### 2.5. Immune Cell Analysis in the Bronchoalveolar Lavage (BALF)

Immediately after the lung function test, saline (0.5 mL, twice) was used to collect the BALF, which was then centrifuged. The cell pellet was re-suspended in 1 mL phosphate buffered saline (PBS). The cell suspension was mixed with 4% Trypan blue (1:1, Life Technologies, Carlsbad, CA, USA). Total cells were then counted using a haemocytometer. Cytospins slides were then made (Thermo Fisher Scientific, Waltham, MA, USA). Then, the slides were fixed and stained with Haem Kwik (Thermo Fisher, Waltham, MA, USA). A differential cell count was performed by counting 4 random fields of view under a light microscope by an observer blinded to the treatment of the mice [22].

### 2.6. Histology 

The severity of peribronchial inflammation was graded using a published method [23] (Grading criteria—0: no immune cells surrounding the airway; 1: a few immune cells surrounding the airway; 2: a ring of cells one cell-layer deep; 3: a ring of cells two cells deep; 4: a ring of cells three to four cells deep; and 5: >4 cells deep). All bronchioles in the images were assessed. The epithelial thickness inside the bronchiole was measured using the distance between the epithelial edge near the lumen and the edge near the bronchiole wall at three non-overlapping locations on each slide by image J. The results were averaged as the value for each biological repeat. The bronchiole diameter was also measured at three non-overlapping locations on each slide and the averaged result was used for each biological repeat. Then, the ratio between the epithelial thickness and the bronchiole diameter was calculated.

The number of mucus-producing goblet cells was identified by Periodic acid–Schiff (PAS) staining. The PAS-positive goblet cell counts were normalised to the length of the bronchial perimeter and expressed as the number of PAS-positive cells per mm of the basement membrane [24].

The Ashcroft score was used to semi-quantify the fibrotic change by Masson’s trichrome staining [25]. The α-smooth muscle actin antibody (1:500, Cell Signalling Technology, Danvers, MA, USA) was used to stain peri-bronchiole α-smooth muscle actin followed by the secondary antibody staining from Horseradish peroxidase anti-rabbit Envision system (Dako Cytochemistry, Tokyo, Japan). The width of alpha-smooth muscle thickness and bronchiole were measured three times per bronchiole and the ratio between alpha-smooth muscle thickness and bronchiole diameters was calculated.

The size of alveoli (mean linear intercept) was measured as previously described [26]. The Newcast computer assisted stereology package (Visiopharm) was used to perform systematic uniform random sampling on 20× magnification of whole-slide images at a sampling density of 50%. Mean linear intercepts were then measured using a custom MATLAB software package with a minimum of 200 counting events per animal. The assessment was scored by an observer blinded to the treatment.

### 2.7. Western Blot

The protein levels of the markers of interest were measured in the lung, including inflammatory markers, mitophagy fusion marker optic atrophy (Opa)-1 (1:2000; Cell Signalling Technology, Danvers, MA, USA), mitophagy fission marker dynamin-related protein (Drp)-1 (1:2000; Cell Signalling Technology, Danvers, MA, USA) and Parkin (1:2000; Cell Signalling Technology, Danvers, MA, USA), following our published methods [27]. Briefly, protein concentration was measured by a DC protein assay and samples were separated in the Ctiterion™TGX Stain-Free Precast Gel (BIO-RAD, Hercules, CA, USA) and then transferred to PVDF membranes (BIO-RAD, Hercules, CA, USA), followed by blocking with 2% BSA. Membranes were incubated with primary antibodies overnight at 4 degrees and incubated with secondary antibodies (1: 10,000, Abcam, Cambridge, UK) for 1 h at room temperature. ChemiDoc (BIO-RAD, Hercules, CA, USA) was used to capture the images and Image J was used to measure band density. β-action was used as a housekeeping protein.

### 2.8. Confocal Microscopy Imaging (Mitochondrial Density and ROS Level)

As it has recently been shown that mitochondrial function can be measured using seahorse assays in frozen tissue [28,29], we measured ROS level and mitochondrial density in flash frozen lung tissue. Confocal laser scanning microscopy images of frozen lung sections were acquired using an Olympus FV3000 confocal laser scanning microscope (Olympus, Japan). All imaging parameters, including laser intensities, Photomultiplier tubes voltage and pinholes, were kept constant during imaging. For total ROS measurement, CellROX Deep Red (Molecular Probes®, ThermoFisher Scientific, Scoresby, VIC, Australia) was used at 5 µM final concentration as validated by several groups [30,31,32,33]. Images were acquired at 633 nm excitation wavelength and detected in the 640–680 nm emission range. MitoTracker Green (200 nM—Molecular Probes®, ThermoFisher Scientific, Scoresby, VIC, Australia), which binds to mitochondrial proteins (free thiol groups of cysteine residues), was used to label mitochondria. MitoTracker Green does not work in fixed tissue as the fixiation changes the nature of the free thiol groups of cysteine, but does label isolated mitochondrial proteins (i.e., does not need a viable cell to work as a label) [34]. Furthermore, this probe has been shown to bind to mitochondrial proteins in frozen tissue by us and others [30,33,34,35]. Images were acquired at 488 nm excitation wavelength and detected in the 510–550 nm emission range [30]. The images for ROS and mitochondrial were overlaid for double staining to measure mitochondrial specific ROS expression. The fluorescence intensity of images was measured by ImageJ. Briefly, three non-overlapping locations within each slide were randomly selected and values were averaged. The intensity was measured by image J (NIH).

### 2.9. In Vitro Mitochondrial Function Assay

In vitro experiments were performed by seeding Beas-2B cells (ATCC, 2 × 10^4^ in each well) and serum-starved overnight. Then, cells were stimulated for 24 h by different concentrations of PM (1, 3 and 10 µg/cm^2^). Oligomycin was used to inhibit ATP synthase. Carbonyl cyanide-p-trifluoromethoxyphenylhydrazone (FCCP) was used to shuttle protons across the mitochondrial inner membrane and induce maximal activity of the electron transport chain. In order to correct the oxygen consumption from non-mitochondrial oxidases, inhibitors of respiratory complex I (Rotenone) and III (Antimycin A) were added at the end of the experiment to stop mitochondrial electron transfer. The concentrations of Oligomycin, FCCP and rotenone with antimycin were 1.5 µM, 1 µM and 0.5 µM, respectively. Mitochondrial respiration was measured by the Seahorse XF24 Extracellular Flux Analyzer and the XF Cell Mito Stress Test kit (Seahorse Bioscience, North Billerica, MA, USA) according to the manufacturer’s instructions. Mitochondrial respiration was reflected by oxygen consumption rates (OCRs).

### 2.10. Statistical Methods

Results are expressed as mean ± SEM. Normality was tested for biological data. If the data were not normally distributed, then they were log-transformed. The data were analysed using one-way or two-way ANOVA, followed by the appropriate post hoc tests (GraphPad Prism 7, Graphpad, CV, USA). Lung function was analysed by two-stage linear step-up procedure of Benjamini, Krieger and Yekutieli, followed by controlling the False Discovery Rate. *p* < 0.05 was considered significant.

## 3. Results

### 3.1. Physical and Chemical Characteristics 

**Figure 2:**
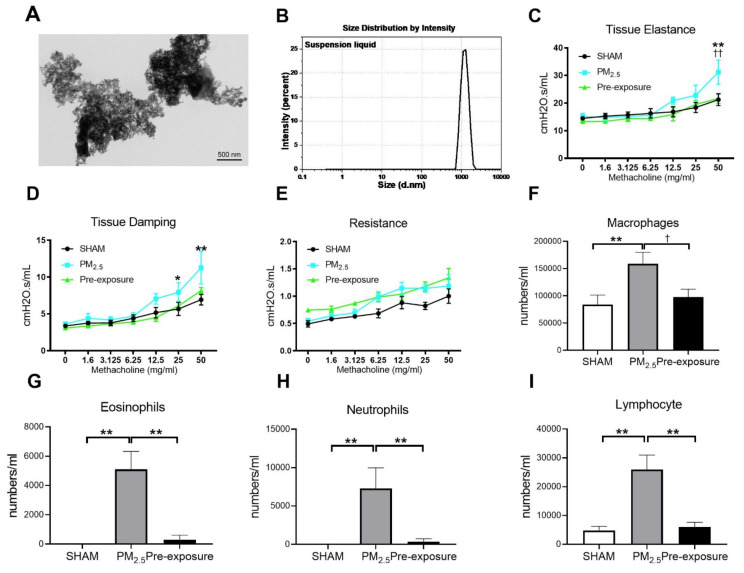
TEM images of PM_2.5_ configuration, body weight, lung function and leukocytes numbers in the dams. Characterisation of the TRAP PM by (**A**) TEM and (**B**) dynamic light scattering. Lung function parameters: (**C**) tissue elastance, (**D**) tissue damping and (**E**) resistance from dams exposed to TRAP PM; the number of (**F**) macrophages, (**G**) eosinophils, (**H**) neutrophils and (**I**) lymphocytes in the dams. Results are expressed as means ± SEM. Data were analysed by one-way ANOVA followed by Fisher’s LSD post hoc tests. Lung function was analysed by the two-stage linear step-up procedure of Benjamini, Krieger, and Yekutieli, followed by controlling the False Discovery Rate. *n* = 8. In (**C**,**D**) * *p* < 0.05, ** *p* < 0.01, SHAM vs. PM_2.5_. †† *p* < 0.01, PM_2.5_ vs. Pre-exposure; in (**F**–**I**), * *p* < 0.05, ** *p* < 0.01, † *p* < 0.05. PM_2.5_: dams exposed to PM_2.5_ (5 µg/day) prior to mating for 6 weeks during gestation and lactation. Pre-exposure: dams exposed to PM_2.5_ for only 6 weeks prior to mating.

The morphology of PM_2.5_ particles was examined by TEM ( Figure 2A). The size of PM ranged from 0.7 to 2 µm as measured by dynamic light scattering (Figure 2B). The main components in the PM_2.5_ were organic carbon, sulphate and elemental carbon (Table 1).

### 3.2. Effects of PM_2.5_ Exposure on the Dams

#### 3.2.1. Low Dose PM_2.5_ Exposure Reduced Body Weight and Lung Function and Increased Leukocyte Numbers in the BALF

PM_2.5_ exposure significantly decreased the body weight of the dams (SHAM 27.2 ± 1.44 g, PM_2.5_ 24.9 ± 0.53 g, *n* = 9, *p* < 0.05). Body weight in the Pre-exposure dams (28.2 ± 2.46 g, *n* = 9) was similar to the SHAM dams. Lung function measured during methacholine challenge showed that the PM_2.5_ dams had increased tissue elastance (*p* < 0.01 vs. SHAM and Pre-exposure groups, Figure 2C), tissue damping (*p* < 0.05 vs. SHAM, Figure 2D) and there were no significant differences between groups in central airway resistance (Figure 2E). In order to determine the impact of PM_2.5_ on pulmonary inflammation, the number of leukocytes in the BALF from the dams were quantified. Immune cell infiltration was examined in the lung. Macrophage (*p* < 0.01 vs. SHAM, *p* < 0.05 vs. Pre-exposure, Figure 2F), eosinophil (*p* < 0.01 vs. SHAM, *p* < 0.01 vs. Pre-exposure, Figure 2G), neutrophil (*p* < 0.01 vs. SHAM, *p* < 0.01 vs. Pre-exposure, Figure 2H) and lymphocyte (*p* < 0.01 vs. SHAM, *p* < 0.01 vs. Pre-exposure, Figure 2I) numbers were significantly bigger in the PM_2.5_ group.

#### 3.2.2. Low Dose PM_2.5_ Exposure Caused Airway Remodelling and Emphysema

**Figure 3:**
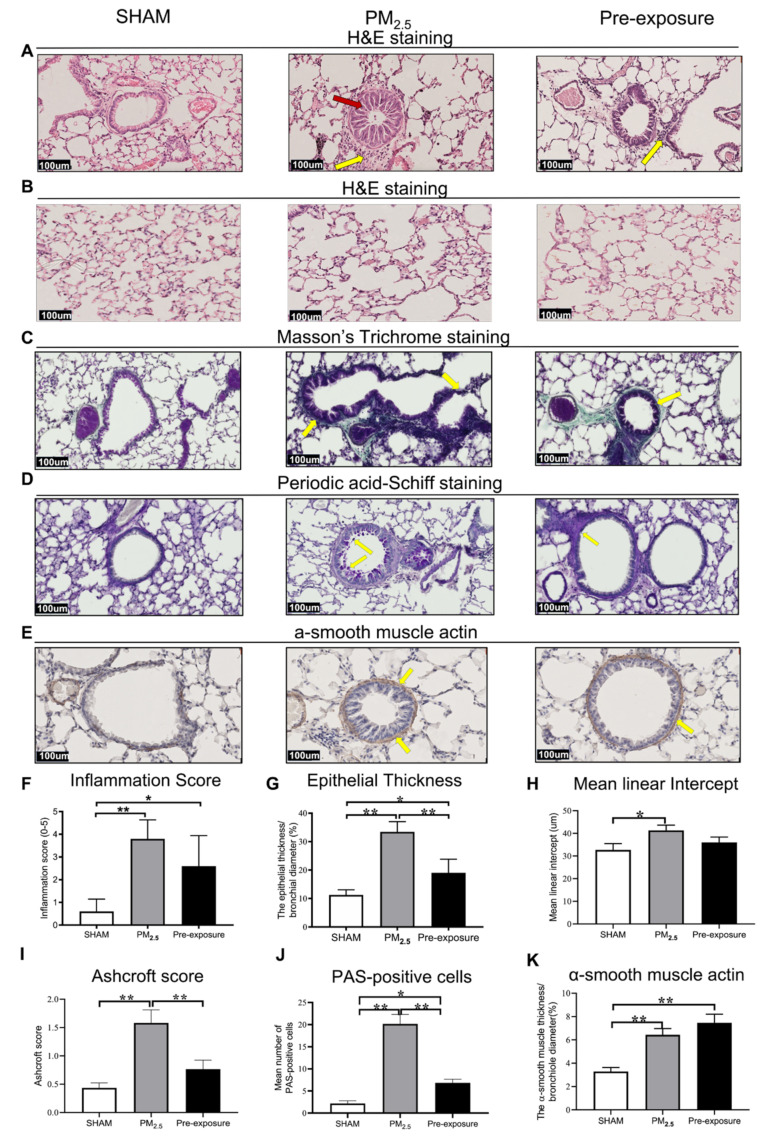
PM_2.5_ exposure induced lung tissue remodelling in the dams. Representative images of lung sections stained with H&E staining of (**A**) the airway (yellow arrows point at immune cells and red arrow point at the enlarged epithelium in the bronchiole) and (**B**) parenchyma; (**C**) Masson’s trichrome staining (yellow arrows point at collagen); (**D**) Periodic acid-Schiff staining, with yellow arrows pointing at goblet cells; and (**E**) α-smooth muscle actin (yellow arrows point at α-smooth muscle actin positive cells). Quantitative results of (**F**) inflammation score, (**G**) epithelial thickness, (**H**) mean linear intercept, (**I**) Ashcroft score, (**J**) PAS-positive cells and (**K**) α-smooth muscle actin. Data were analysed by one-way ANOVA followed by Fisher’s LSD post hoc tests. *n* = 8. * *p* < 0.05, ** *p* < 0.01. PM_2.5_: dams exposed to PM_2.5_ (5 µg/day) prior to mating for 6 weeks during gestation and lactation. Pre-exposure: dams exposed to PM_2.5_ for 6 weeks prior to mating.

Histological analysis revealed epithelial cell hypertrophy in the bronchioles of the PM_2.5_ and Pre-exposure dams (Figure 3A). More immune cells were found around the airways in the PM_2.5_ (*p* < 0.01 vs. SHAM, Figure 3A,F) and Pre-exposure dams (*p* < 0.05 vs. SHAM, Figure 3A,F). The epithelial thickness was significantly greater in airways from both PM_2.5_ (*p* < 0.01 vs. SHAM, Figure 3A,G) and Pre-exposure dams (*p* < 0.05 vs. SHAM, Figure 3A,G). Alveolar enlargement and parenchymal simplification were found in the PM_2.5_ dams (Figure 3B) and the mean linear intercept measurement (a measurement of the amount of emphysema) was significantly increased in PM_2.5_ dams (*p* < 0.05 vs. SHAM, Figure 3H). Increased collagen deposition in airways was evident in the sections stained by Masson’s Trichrome in the PM_2.5_ dams (*p* < 0.01 vs. SHAM, *p* < 0.01 vs. Pre-exposure, Figure 3C,I). This was accompanied by mucus hyper-secretion in both PM_2.5_ (*p* < 0.01 vs. SHAM, *p* < 0.01 vs. Pre-exposure, Figure 3D,J) and Pre-exposure dams (*p* < 0.05 vs. SHAM, Figure 3D,J). Higher α-smooth muscle actin expression around the airway was found in the PM_2.5_ and Pre-exposure dams (both *p* < 0.01 vs. SHAM, Figure 3E,K).

#### 3.2.3. PM_2.5_ Exposure Increased Total ROS, Dynamin-Related Protein (Drp)-1 and Parkin Levels

**Figure 4:**
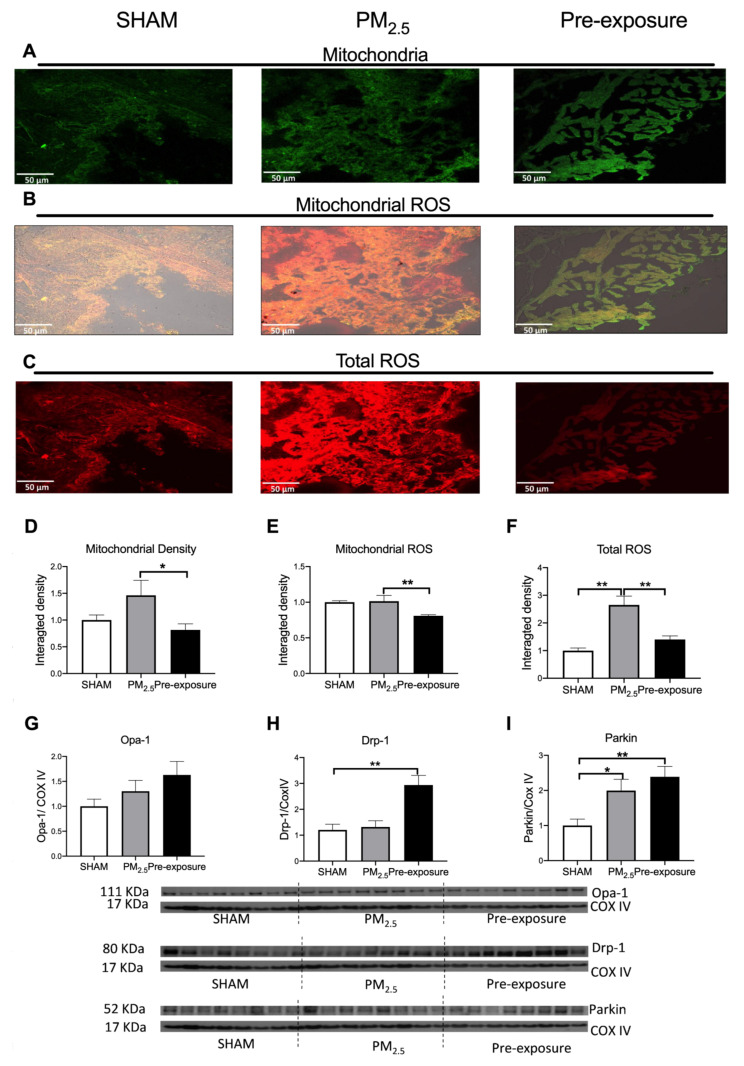
PM_2.5_ exposure increased mitochondrial density and total ROS level in the dams. Representative images of (**A**) mitochondria (green), (**B**) mitochondrial specific ROS (orange,) and (**C**) total ROS (red) staining, and (**D**–**F**) quantitative results. Protein levels of (**G**) Opa-1, (**H**) Drp-1 and (**I**) Parkin. Data were analysed by one-way ANOVA followed by Fisher’s LSD post hoc tests. N = 5 in mitochondrial fluorescence staining, *n* = 8 in Western blot. * *p* < 0.05, ** *p* < 0.01. Drp-1, dynamin-related protein; Opa-1, optic atrophy-1; PM_2.5_: dams exposed to PM_2.5_ (5 µg/day) prior to mating for 6 weeks during gestation and lactation. Pre-exposure: dams exposed to PM_2.5_ for 6 weeks prior to mating.

There was a trend towards increased mitochondrial density in PM_2.5_ dams’ lung, which resulted in a similar ROS level per mitochondrion in the PM_2.5_ dams compared to the SHAM dams (Figure 4A,B,D,E). However, the total ROS level in lung tissues was significantly higher in the PM_2.5_ dams (*p* < 0.01 vs. SHAM and Pre-exposure, Figure 4C,F). There were no significant differences in the mitophagy fusion marker optic atrophy 1 (Opa-1) level between the groups, although an increasing trend can be found after PM exposure (Figure 4G). Compared to the SHAM dams, the mitophagy fission marker Drp-1 level was significantly increased in the Pre-exposure dams (*p* < 0.01 vs. SHAM, Figure 4H). The levels of Parkin, another fission marker that labels damaged mitochondria, in PM_2.5_ (*p* < 0.05 vs SHAM, Figure 4I) and Pre-exposure dams (*p* < 0.01 vs. SHAM, Figure 4I) were significantly higher than the SHAM dams.

### 3.3. Effects of Maternal PM_2.5_ Exposure on Offspring at 13 Weeks 

#### 3.3.1. Maternal PM_2.5_ Exposure Decreased Litter Size and Influenced Body Weight

The average litter size in the SHAM group and a sex ratio close to 1 were expected for this mouse strain. The litter sizes of PM_2.5_ exposed dams were smaller compared to that of the SHAM dams, with more females in each litter (Table 2), suggesting survival pressure. Maternal chronic exposure to low dose PM_2.5_ significantly decreased the body weight of female offspring at 13 weeks (*p* < 0.05 PM_2.5_ group vs. SHAM, Table 2). In the Pre-exposure group, litter size, sex ratio and offspring’s body weight were similar to the SHAM group. There were no significant differences in body weight among three groups in the male offspring (Figure S1).

#### 3.3.2. Maternal PM_2.5_ Exposure Resulted in Airway Inflammation

**Figure 5:**
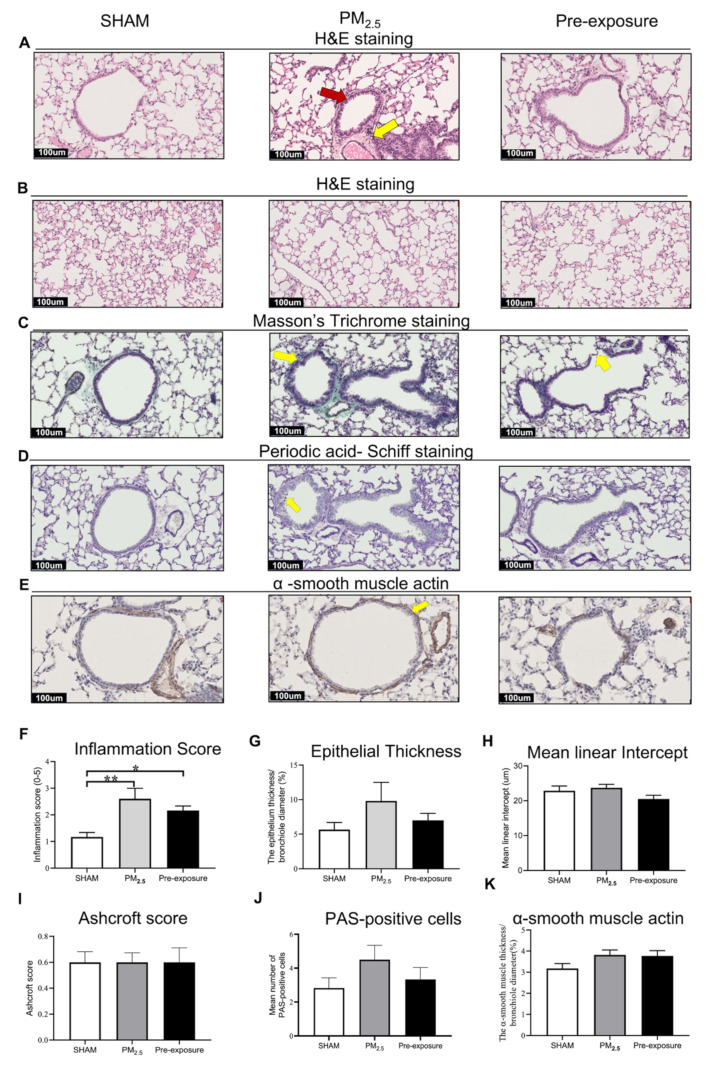
Maternal PM_2.5_ exposure increased inflammation in the female offspring at 13 weeks. Representative images of lung sections stained with (**A**) H&E staining of the airway (yellow arrow point at immune cells and red arrow point at enlarged epithelium in the bronchiole) and (**B**) parenchyma; (**C**) Masson’s trichrome staining (yellow arrows pointed at the collagen); (**D**) Periodic acid-Schiff staining (yellow arrow point at goblet cells); and (**E**) α-smooth muscle actin (yellow arrows point at α-smooth muscle actin). Quantitative results of (**F**) inflammation score, (**G**) epithelial thickness, (**H**) mean linear intercept, (**I**) Ashcroft score, (**J**) PAS-positive cells and (**K**) α-smooth muscle actin. Data were analysed by one-way ANOVA followed by Fisher’s LSD. *n* = 8. * *p* < 0.05, ** *p* < 0.01. PM_2.5_: dams exposed to PM_2.5_ (5 µg/day) prior to mating for 6 weeks during gestation and lactation. Pre-exposure: dams exposed to PM_2.5_ for only 6 weeks prior to mating.

Histological analysis revealed that both female and male offspring with *in-utero* PM_2.5_ exposure had increased inflammatory cells in the lungs (Figure 5A,F and Figure S2A,F). The inflammation score was also increased in both female and male offspring from the PM_2.5_ (*p* < 0.01 vs. SHAM, Figure 5F and Figure S2A) and Pre-exposure dams (*p* < 0.05 vs. SHAM, Figure 5F and Figure S2F). There were no significant changes in epithelial thickness (Figure 5A,G and Figure S2A,G), alveolar size (Figure 5B,H and Figure S2B,H), Ashcroft score (a measure of fibrosis, Figure 5C,I and Figure S2C,I), PAS-positive cells (Figure 5D,J and Figure S2D,J) or airway smooth muscle content (Figure 5E,K and Figure S2E,K) in any sex.

#### 3.3.3. Maternal PM_2.5_ Exposure Increased Mitochondrial Density, ROS and Drp-1 Levels and Decreased Parkin Level

**Figure 6:**
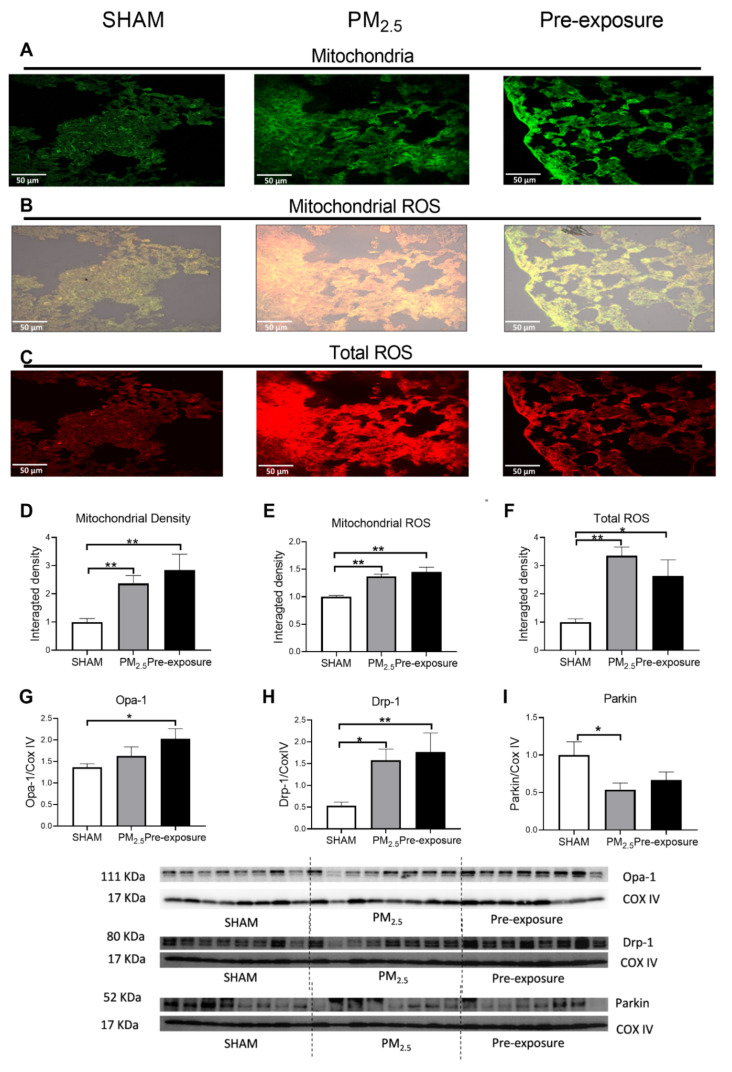
Maternal PM_2.5_ exposure changed mitochondrial density and ROS level in the female offspring at 13 weeks. Representative images of (**A**) mitochondria (green), (**B**) mitochondrial specific ROS (orange) and (**C**) total ROS staining (red), and (**D**–**F**) quantitative results. Protein levels of (**G**) Opa-1, (**H**) Drp-1 and (**I**) Parkin. Data were analysed by one-way ANOVA followed by Fisher’s LSD post hoc tests. * *p* < 0.05, ** *p* < 0.01, *n* = 5 in mitochondrial fluorescence staining, *n* = 8 in Western blot. Drp-1, dynamin-related protein; Opa-1, optic atrophy-1; PM_2.5_: dams exposed to PM_2.5_ (5 µg/day) prior to mating for 6 weeks during gestation and lactation. Pre-exposure: dams exposed to PM_2.5_ for 6 weeks prior to mating.

Mitochondrial density and mitochondrial specific ROS levels were significantly higher in both the PM_2.5_ (*p* < 0.01 vs. SHAM, Figure 6A,B,D,E) and the Pre-exposure offspring (*p* < 0.01 vs. SHAM, Figure 6A,B,D,E) in female offspring. Compared to the SHAM group, total ROS levels were also increased in the PM_2.5_ (*p* < 0.01 vs. SHAM, Figure 6C,F) and Pre-exposure offspring (*p* < 0.05 vs. SHAM, Figure 6 C,F) in female offspring. In male offspring, the mitochondrial density was also significantly increased in the PM_2.5_ group (*p* < 0.05 vs. SHAM, Figure S3A,D). However, mitochondrial ROS and total ROS levels were similar among the three male groups (Figure S3B,C,E,F).

In female offspring, the fusion marker Opa-1 level in the Pre-exposure group was significantly higher than that in the SHAM group (*p* < 0.05 vs. SHAM, Figure 6G). Both PM_2.5_ (*p* < 0.05 vs. SHAM) and Pre-exposure (*p* < 0.01 vs. SHAM) groups have higher fission marker Drp-1 levels (Figure 6H). Parkin level was significantly reduced in the PM_2.5_ group (*p* < 0.05 vs. SHAM, Figure 6I). In male offspring, the Drp-1 level was significantly decreased in the Pre-exposure group (*p* < 0.01 vs. SHAM and PM_2.5_, Figure S3H). There was no significant difference in the Opa-1 and Parkin levels between groups in males (Figure S3G,I).

#### 3.3.4. Maternal PM_2.5_ Exposure Caused Intrinsic Airways Hyperresponsiveness in Female Offspring and Leukocytes Numbers in the BALF of Both Sexes

**Figure 7:**
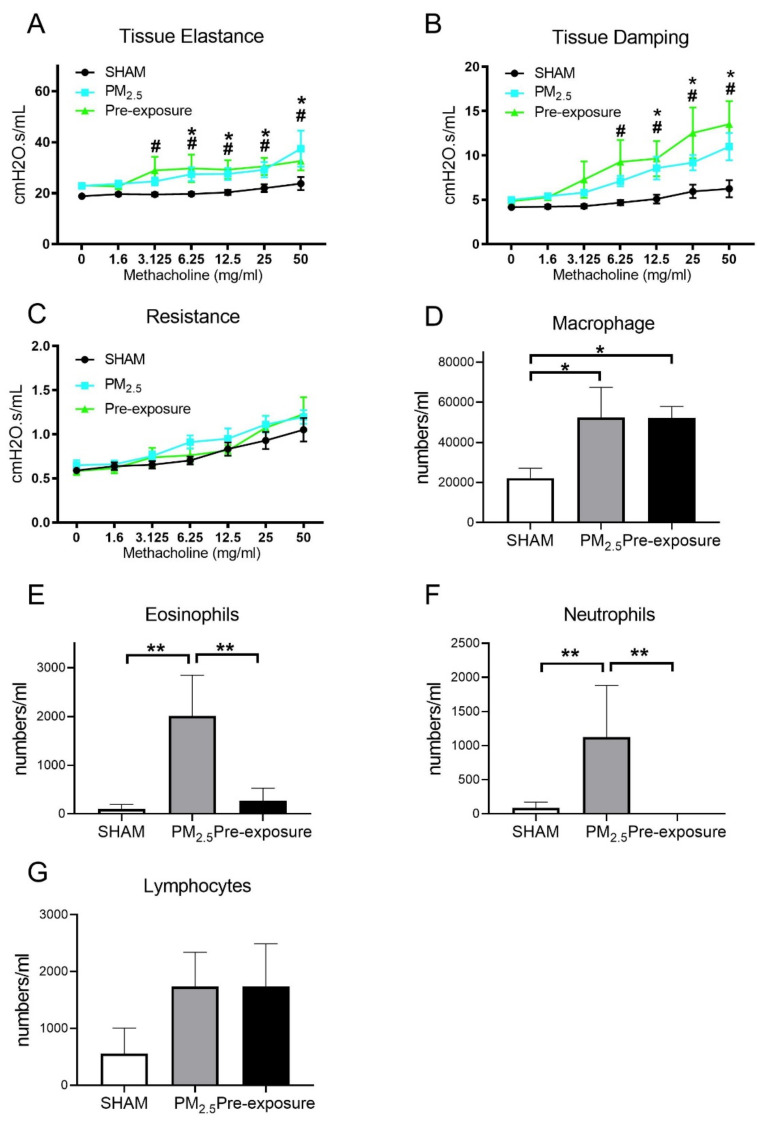
Lung function and leukocytes numbers in the female offspring at 13 weeks. Lung function of (**A**) tissue elastance, (**B**) tissue damping, (**C**) resistance, and the number of (**D**) macrophages, (**E**) eosinophils, neutrophils (**F**) and lymphocytes (**G**) in the dams. Results are expressed as means ± SEM. Data were analysed by one-way ANOVA followed by Fisher’s LSD post hoc tests, lung function was analysed by the Two-stage linear step-up procedure of Benjamini, Krieger, and Yekutieli followed by controlling the False Discovery Rate. *n* = 8. In (A,B) * *p* < 0.05, ** *p* < 0.01, SHAM vs. PM_2.5_. ^#^ *p* < 0.05, SHAM vs. Pre-exposure. In (**D**–**F**) * *p* < 0.05, ** *p* < 0.01. PM_2.5_: dams exposed to PM_2.5_ (5 µg/day) prior to mating for 6 weeks during gestation and lactation. Pre-exposure: dams exposed to PM_2.5_ for 6 weeks prior to mating.

During the lung function test, in response to the methacholine challenge, the female offspring from PM_2.5_ exposed dams had increased tissue elastance (*p* < 0.05, PM_2.5_ vs. SHAM, Figure 7A) and tissue damping (*p* < 0.05, PM_2.5_ vs. SHAM, Figure 7B). These findings were mirrored in the offspring from the Pre-exposure group. There were no significant differences in resistance between the three female offspring groups (Figure 7C).

Immune cell infiltration was examined in the lung. Macrophage (*p* < 0.05 vs. SHAM, Figure 7D), eosinophil (*p* < 0.01 vs. SHAM, *p* < 0.01 vs. Pre-exposure, Figure 7E) and neutrophil (*p* < 0.01 vs. SHAM, *p* < 0.01 vs. Pre-exposure, Figure 7F) numbers were significantly bigger in the female offspring from PM_2.5_ dams. Macrophage numbers in female offspring from the Pre-exposure dams were also significantly bigger than the SHAM offspring (*p* < 0.05 vs. SHAM, Figure 7D). There were no significant differences in lymphocyte numbers between groups (Figure 7G). In the male offspring, the macrophage (*p* < 0.01 vs. SHAM, *p* < 0.05 vs. Pre-exposure, Figure S4A) and neutrophil (*p* < 0.05 vs. SHAM, Figure S4C) numbers were significantly bigger in the PM_2.5_ groups. There were no significant differences in eosinophils (Figure S4B) and lymphocytes (Figure S4D) in the BALF among the male groups, although eosinophils only seemed to be markedly higher in the male offspring in the PM_2.5_ groups.

#### 3.3.5. Maternal PM_2.5_ Exposure Increased Airway Hyperresponsiveness (AHR) in OVA Sensitised Animals

**Figure 8:**
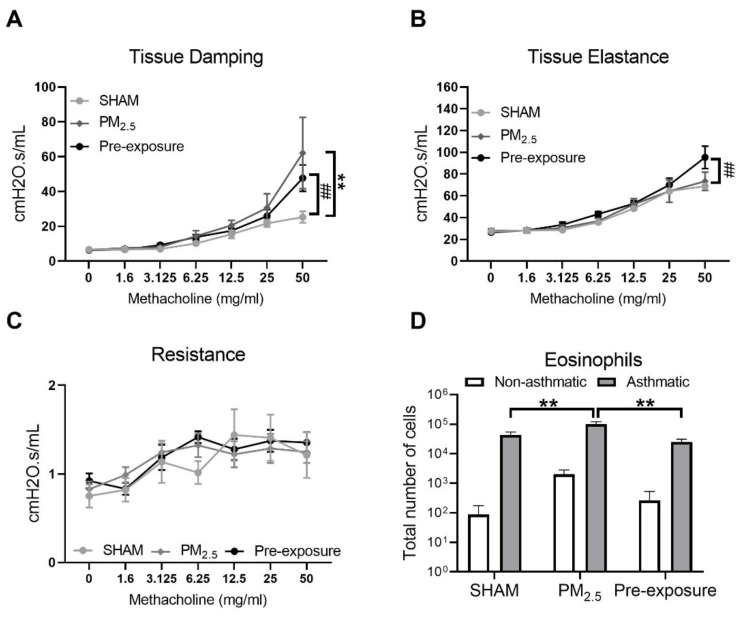
Maternal PM_2.5_ exposure increased AHR in OVA sensitised animals. (**A**) Lung function of tissue elastance, (**B**) tissue damping and (**C**) resistance, and (**D**) eosinophil number in the BALF in response to OVA in the female offspring at 13 weeks. Data were analysed by two-way ANOVA (mixed-model) followed by Fisher’s LSD post hoc tests. Lung function was analysed by the two-stage linear step-up procedure of Benjamini, Krieger, and Yekutieli, followed by controlling the False Discovery Rate. *n* = 8. In (A, B) ** *p* < 0.01, PM_2.5_ vs. SHAM. ## *p* < 0.01, Pre-exposure vs. SHAM. In (**D**), ** *p* < 0.01. PM_2.5_: mice exposed to PM_2.5_ (5 µg/day) prior to mating for 6 weeks during gestation and lactation. Pre-exposure: PM_2.5_ exposure 6 weeks prior to mating. SHAM (OVA): offspring from SHAM dams challenged with OVA. PM_2.5_ (OVA): offspring from PM_2.5_ dams challenged with OVA. Pre-exposure (OVA): offspring from Pre-exposure dams challenged with OVA.

Since female offspring with in utero PM_2.5_ exposure displayed some features consistent with the hallmarks of human asthma (AHR and inflammation within the airways), a separate cohort of female offspring (+/− in utero PM_2.5_ exposure) was exposed to OVA to model the development of allergic airways disease (asthma). Increased airway responsiveness was found reflected by the changes in tissue damping (*p* < 0.01 PM_2.5_ vs. SHAM, *p* < 0.01 Pre-exposure vs. SHAM, Figure 8A,B) and tissue elastance (*p* < 0.01 Pre-exposure vs. SHAM, Figure 8B). Tissue resistance was not different between groups (Figure 8C). An increased number of eosinophils in the BALF was found in the PM_2.5_ group (*p* < 0.01 vs. SHAM and Pre-exposure, Figure 8D).

### 3.4. PM Exposure Reduced Mitochondrial Respiration Which Was Reversed by Mitochondria-Targeted Antioxidant Ubiquinone (MitoQ) Treatment In Vitro

**Figure 9:**
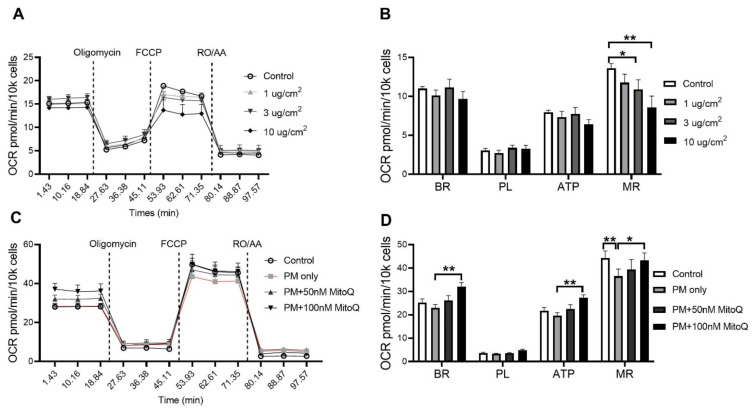
PM exposure reduced mitochondrial respiration in the BEAS-2B cells which was reversed by MitoQ. (**A**,**B**) Mitochondrial respiration in the BEAS-2B cells after PM exposure with 1 µg/cm^2^, 3 µg/cm^2^ and 10 µg/cm^2^ followed by (**C**,**D**) MitoQ treatment. Data were analysed by one-way ANOVA followed by Fisher’s LSD post hoc tests. * *p* < 0.05, ** *p* < 0.01. FCCP: carbonyl cyanide-p-trifluoromethoxyphenylhydrazone; RO: Rotenone; AA: Antimycin A; OCR: Oxygen consumption rate; ATP: ATP-linked Respiration; BR: Basal Respiration; MR: Maximal Respiration; PL: Proton Leak-linked Respiration; PM: Particulate matter.

In order to investigate the effect of PM exposure on mitochondrial respiration, oxygen consumption rate (OCR) was measured in vitro. The BEAS-2B epithelial cell line was stimulated for 24 h with various concentrations of PM_2.5_ (1, 3 and 10 µg/cm^2^, Figure 9A). A significant reduction in the maximal respiration (MR) was found after PM stimulation at 3 µg/cm^2^ (*p* < 0.05 vs. Control, Figure 9B) and 10 µg/cm^2^ (*p* < 0.01 vs. Control, Figure 9B). In order to determine whether this functional impairment was due to increased mitochondrial oxidative stress, cells were treated with mitochondrial-targeted antioxidant MitoQ (100 nM pre-determined in the same cells) when exposed to PM_2.5_. MitoQ significantly reversed mitochondrial dysfunction induced by PM exposure at 10 µg/cm^2^ (Figure 9C,D). Compared to the PM only group, significant increases in basal respiration (BR) (*p* < 0.01 vs. PM only, Figure 9D), ATP-linked respiration (ATP) (*p* < 0.01 vs. PM only, Figure 9C,D) and maximal respiration (MR) (*p* < 0.05 vs. PM only, Figure 9D) were found after MitoQ treatment. There were no significant differences in proton leak-linked respiration (PL) among the groups (Figure 9C,D).

## 4. Discussion

The novelty of this study can be divided into four findings. Firstly, in the dams, chronic exposure to low dose PM_2.5_ (representative of clean air countries) resulted in emphysema-like pathology, reflected by lung parenchymal damage, airway hyperresponsiveness, leukocyte infiltration and mitochondrial disorders. Secondly, for the first time, we show that even the inhalation of low dose PM during pregnancy can cause adverse lung health outcomes in female and male offspring reflected by chronic lung inflammation and female offspring had intrinsic airway hyper-responsiveness and an enhanced response to allergic airways disease. Thirdly, we observed a significant sex difference in response to in utero PM exposure, where female offspring’s lungs were more affected than their male littermates. Fourthly, maternal PM exposure before pregnancy can also damage lung health in female offspring, but not the males.

Low levels of air pollution are often regarded as not being harmful and can lead to the construction of residential buildings near busy roads, such as in Sydney, which will require 664,000 more homes to house the extra 1.6 million people by 2035. One explicit strategy for achieving this is to increase housing stock along transport corridors which will expose more people to pollution [36]. The previous study found that a higher concentration of PM_2.5_ exposure could increase the rate of low birth weight (LBW). The average PM_2.5_ concentration in New York was 13 μg/m^3^, which resulted in 2.8% of LBW; however, the PM_2.5_ levels are lower in Minnesota (9 μg/m^3^), which only induced 1.9% of LBW [37]. The unchanged birth weight in PM offspring shown in this study is not surprising given the PM dose in our study was only 5 μg/day.

Inhalation of air pollution and, in particular, high levels of air pollution is associated with a range of respiratory diseases and, in particular, asthma and chronic obstructive pulmonary disease (COPD), which is a pattern that mirrors the effects of cigarette smoking. The timing of exposure and effects in children and adults are, however, different. In an otherwise healthy individual, chronic long-term exposure to air pollution will cause lung injury and induce COPD in susceptible people [38]. If exposure occurs during pregnancy, there are detrimental perinatal outcomes, including decreased birth weight and an increased risk of several chronic diseases which includes asthma [39].

In our study, we found that maternal chronic exposure to low dose TRAP PM caused lung injury, inflammation and AHR in both dams and female offspring, whereas male offspring only showed increased lung inflammation without measurable lung structural damage. The mechanisms driving the changes in lung physiology in dams and offspring are likely to be different. Here, the most likely explanation for the increased airway reactivity to methacholine in dams is the loss of alveolar attachments and the increase in smooth muscle and airway wall thickening. Alveoli attach to the airways and assist in maintaining patency (i.e., they keep the airways open), whilst the combination of increased muscle and airway wall size renders the airways more reactive or more likely to narrow to the point of closure. Methacholine challenged dams in the PM_2.5_ group exhibited increased tissue elastance and tissue damping, which is consistent with damage to the airway and lung parenchyma. These results are consistent with one previous study using combustion derived ultrafine particles containing persistent free radicals; however, that dose was 10 times higher than the one in our study [15]. Those features in the dams are consistent with the characteristics of COPD, including thicker small airway walls, greater epithelial cell hyperplasia, enhanced squamous metaplasia, collagen deposition and mucus hypersecretion. Theoretical models based on patients have demonstrated that damaged alveoli (emphysema) increases lung elastance in patients with emphysema, followed by higher tissue damping [40]. Clinical data published in our group also supported increased elastance in COPD patients [41]. Interestingly, as it occurs in humans, tissue remodelling did not resolve following the removal of the exposure as inflammation and remodelling still occurred in the Pre-exposure group. Taken together, these data suggests that living in an environment with a low level of TRAP PM pollution may increase the risk of developing COPD.

High dose *in-utero* PM exposure has been shown to cause growth retardation, poor lung development/remodelling and inflammation in murine models [15]. In the current study with low dose PM exposure, we also found AHR in both PM_2.5_ and Pre-exposure female offspring only, accompanied by increased inflammation, mitochondrial density and ROS level. These features suggest that maternal low dose TRAP PM_2.5_ exposure increases the risk of respiratory disease in the female offspring, with risks starting even before pregnancy. Epidemiological studies have found that maternal PM_2.5_ exposure was associated with a higher risk of developing asthma [39]. In order to further investigate this, we used a classical model of allergic airways disease (asthma) [42], the OVA model, and found that female offspring with in utero TRAP PM_2.5_ exposure had enhanced eosinophilia and AHR. In the Pre-exposure group, it was surprising that only six weeks of PM exposure before pregnancy (i.e., not during gestation) also affected the offspring. This suggests that the effects of TRAP PM_2.5_ exposure extend well beyond the actual period of exposure, but this observation requires further investigation.

We speculate that mitochondrial dysfunction due to PM_2.5_ exposure is transferable from the mothers to the offspring since mitochondria are exclusively inherited from the mothers. The fission marker Drp-1 was increased in the dams indicating impaired mitochondrial division. This will decrease the connectivity and the size of mitochondria, resulting in mitochondrial dysfunction [43], which is further confirmed by the upregulation of Parkin [43]. Mitochondrial dysfunction was also found in the female offspring and this is reflected by the changes in Drp-1 and Parkin in their lungs. Increased Drp-1 and decreased Parkin levels reflect the imbalance between mitochondrial dynamics, which likely contribute to excessive mitochondrial ROS generation [44]. Typically during PM exposure, phagocytes such as macrophages and neutrophils can produce excessive ROS and result in tissue injury [45,46]. In our study, we found increased ROS consistent with significantly bigger macrophage and neutrophil numbers and tissue remodelling in both PM exposed dams and female offspring. The resulting lung changes could result in the future development of COPD and also fetal growth restriction affecting early lung development [44,47].

Recent studies also demonstrated that inhaled airborne PM can cross the placenta [48]. In our study, we did not observe any evidence of PM in alveolar macrophages with in utero exposure, however, we cannot exclude the possibility that PM directly affects the developing fetus. In order to further confirm the role of ROS in our model, MitoQ, a mitochondria-targeted antioxidant, was used to increase the capacity of ROS scavenging. OCR results showed that MitoQ treatment could significantly reverse the adverse impacts induced by PM exposure. This is consistent with a previous mouse study in which MitoQ supplementation could reverse lung injury by ozone exposure [49]. These results highlight that mitochondrial ROS induced by maternal PM exposure is one of the principal mechanisms by which lung injury occurs.

Our study does have limitations. We observed a significant sex difference, with female offspring being more affected; however, we do not know why male offspring were protected from underdevelopment and lung structural injury by in utero PM exposure. This warrants further investigation, including the lung function measurement in both sexes. In addition, we did not investigate the development of the lungs during early life, which may provide the mechanism of why maternal exposure to the low-level PM could induce lung dysfunction in female offspring but not in the males. Increased cell proliferation and apoptosis have been suggested to contribute to airway remodelling in asthmatic lungs [50]. Future studies can include these aspects to determine whether in utero PM exposure induced remodelling is, in part, the result of hyperplasia and/or apoptosis. Furthermore, we did not investigate if gametes from the dams are epigenetically modified and if these changes are preserved during fertilisation and in adult offspring. These need to be followed up in future studies.

## 5. Conclusions

Chronic exposure to low dose PM_2.5_ can cause lung damage in the dams, which is consistent with the pathology of COPD. Moreover, maternal PM_2.5_ exposure can have long-lasting detrimental effects on lung function and increase the risk of asthma in the offspring. Removing PM exposure during pregnancy did not provide complete protection to either the dams or the offspring. Mitochondrial dysfunction and oxidative stress are plausible mechanisms by which these adverse impacts occur.

## Figures and Tables

**Table 1:** Chemical characteristics of PM_2.5_.

Component Quantity	ug/m^3^
Chloride	0.44 ± 0.03
Nitrite	0.2 ± 0.01
Nitrate	3.11 ± 0.25
Sulphate	8.29 ± 0.48
Sodium	2.62 ± 0.14
Ammonium	1.71 ± 0.13
Potassium	0.24 ± 0.02
Organic carbon	7.86 ± 0.51
Elemental carbon	4.46 ± 0.20

The results were expressed as ± SEM. *n* = 10.

**Table 2:** Litter size, sex ratio and body weight of female offspring.

	Litter Size (*n*)	Sex Ratio (M/F)	Body Weight (g)
SHAM	5 ± 1.47	1.14	21.74 ± 1.91
PM_2.5_	4.66 ± 2.35	0.83	20.11 ± 1.88 *
Pre-exposure	5 ± 2.90	1.02	20.78 ± 0.59

Results are expressed as mean ± SEM and were analysed by one-way ANOVA followed by Fisher’s LSD post hoc tests. *n* = 10. * *p* < 0.05 PM_2.5_ vs. SHAM. PM_2.5_: maternal PM_2.5_ exposure prior to mating for 6 weeks during gestation and lactation. Pre-exposure: maternal exposed to PM_2.5_ for 6 weeks prior to mating.

## Data Availability

All data are included in this paper and in the Supplementary Materials.

## Supplementary Materials

The following are available online at https://www.mdpi.com/article/10.3390/antiox10071029/s1. Figure S1: The body weight of male offspring at 13 weeks. Figure S2: Maternal PM_2.5_ exposure increased inflammation in the male offspring at 13 weeks. Figure S3: Maternal PM_2.5_ exposure changed mitochondrial density in the male offspring at 13 weeks. Figure S4: The leukocyte numbers in the male offspring at 13 weeks.

## Abbreviations

AHR: airways hyper-responsiveness; BALF: bronchoalveolar lavage; Drp: dynamin-related protein; Opa: optic atrophy; OVA: ovalbumin; OCRs: oxygen consumption rates; FCCP: carbonyl cyanide-p-trifluoromethoxyphenylhydrazone; RO: Rotenone; AA: Antimycin A; BR: basal respiration; MR: and maximal respiration; PL: proton leak-linked respiration; PAS: Periodic acid–Schiff; PM: particulate matter; ROS: reactive oxygen species; TRAP: traffic-related air pollution; TEM: transmission electron microscope.
