# Supplementary material for: Maternal Particulate Matter Exposure Impairs Lung Health and Is Associated with Mitochondrial Damage
Source: Antioxidants (Basel). 2021 Jun 25;10(7):1029. doi: 10.3390/antiox10071029 (PMC8300816; doi:10.3390/antiox10071029)
Supplement: Supplementary file 1 [file antioxidants-10-01029-s001.zip › antioxidants-1263669-supplementary.pdf]

antioxidants
MDPI

Article

Maternal Particulate Matter Exposure Impairs Lung Health and Is Associated with Mitochondrial Damage

Baoming Wang [1,2], Yik Lung Chan [1,2], Gerard Li [1], Kin Fai Ho [3], Ayad G. Anwer [4], Bradford J. Smith [5], Hai Guo [6], Bin Jalaludin [7,8], Cristan Herbert [9], Paul S. Thomas [9], Jiayan Liao [10], David G. Chapman [1,2], Paul S. Foster [11], Sonia Saad [12], Hui Chen [1,†] and Brian G Oliver [1,2,*,†]

[1] Faculty of Science, School of Life Sciences, University of Technology Sydney, Ultimo, NSW 2007, Australia; Baoming.Wang@student.uts.edu.au (B.W.); Yik.chan@uts.edu.au (Y.L.C.); gerard.E.li@student.uts.edu.au (G.L.); David.Chapman@uts.edu.au (D.G.C.); hui.chen-1@uts.edu.au (H.C.)
[2] Respiratory Cellular and Molecular Biology, Woolcock Institute of Medical Research, The University of Sydney, NSW 2037, Australia
[3] Jockey Club School of Public Health and Primary Care, The Chinese University of Hong Kong, Hong Kong, China; kfho@cuhk.edu.hk
[4] ARC Centre of Excellence for Nanoscale Biophotonics, Faculty of Engineering, Graduate School of Biomedical Engineering, UNSW Sydney, Sydney, NSW 2052, Australia; a.anwer@unsw.edu.au
[5] Department of Bioengineering, Department of Paediatric Pulmonary and Sleep Medicine, School of Medicine, University of Colorado, Boulder, CO 80309, USA; Bradford.smith@cuanschutz.edu
[6] Air Quality Studies, Department of Civil and Environmental Engineering, Hong Kong Polytechnic University, Hong Kong, China; ceguohai@polyu.edu.hk (H.G.)
[7] Ingham Institute for Applied Medical Research, University of New South Wales, Sydney, NSW 2052, Australia; b.jalaludin@unsw.edu.au
[8] Centre for Air Pollution, Energy and Health Research (CAR), Woolcock Institute of Medical Research, The University of Sydney, NSW 2037, Australia
[9] Department of Pathology, Faculty of Medicine, School of Medical Sciences, and Prince of Wales' Clinical School, University of New South Wales, Sydney, NSW 2052, Australia; c.herbert@unsw.edu.au (C.H.); paul.thomas@unsw.edu.au (P.S.T.)
[10] Institute for Biomedical Materials and Devices, Faculty of Science, University of Technology Sydney, Ultimo, NSW 2007, Australia; Jiayan.Liao@student.uts.edu.au
[11] Priority Research Centre for Healthy Lungs, University of Newcastle, Callaghan, NSW 2308, Australia; Paul.Foster@newcastle.edu.au
[12] Renal Group, Kolling Institute of Medical Research, The University of Sydney, St Leonards, Sydney, NSW 2064, Australia; Sonia.saad@sydney.edu.au
* Correspondence: brian.oliver@uts.edu.au
† joint senior authors.

Supplementary Materials

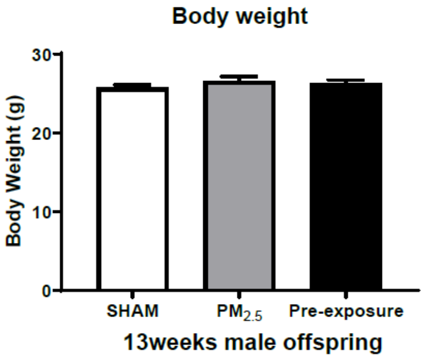

Figure S1: The body weight of male offspring at 13 weeks. Results are expressed as mean ± SEM and were analysed by one-way ANOVA followed by Fisher's LSD post hoc tests. n = 10. PM2.5: maternal PM2.5 exposure prior to mating for 6 weeks, during gestation and lactation. Pre-exposure: maternal exposed to PM2.5 for 6 weeks prior to mating.

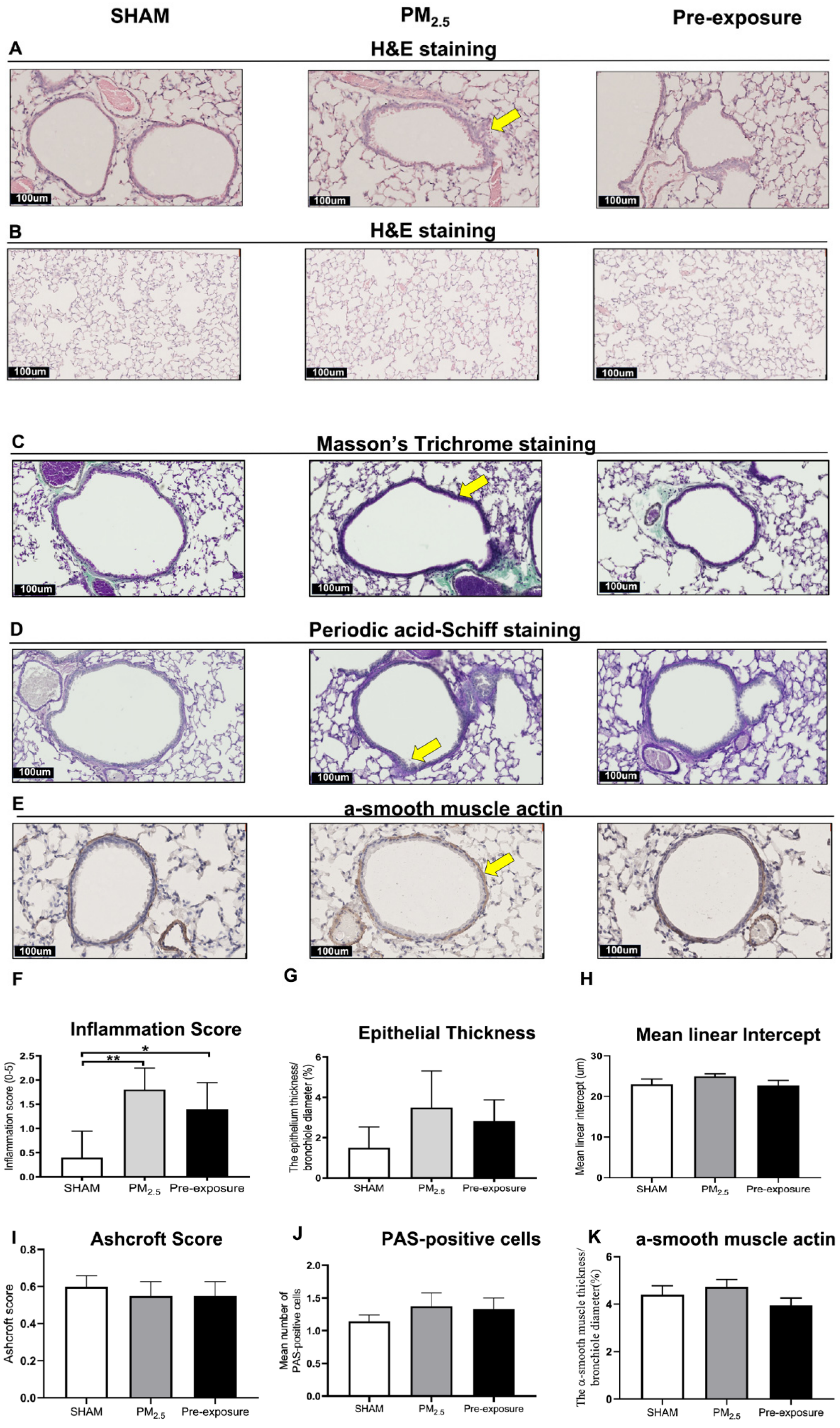

Figure S2. Maternal PM2.5 exposure increased inflammation in the male offspring at 13 weeks. Representative images of lung sections stained with (A) H&E staining of the airway (yellow arrow point at immune cells) and (B) parenchyma; (C) Masson's trichrome staining (yellow arrow point at collagen), (D) Periodic acid-Schiff staining (yellow arrow point at

goblet cells), and (E) α-smooth muscle actin (yellow arrow point at α-smooth muscle actin). Quantitative results of (F) inflammation score, (G) epithelial thickness, (H) mean linear intercept, (I) Ashcroft score, (J) PAS-positive cells and (K) α-smooth muscle actin. Data were analysed by one-way ANOVA followed by Fisher's LSD. N=8. *$p < 0.05$, **$p < 0.01$. $PM_{2.5}$: dams exposed to $PM_{2.5}$ (5μg/day) prior to mating for 6 weeks, during gestation and lactation. Pre-exposure : dams exposed to $PM_{2.5}$ for only 6 weeks prior to mating.

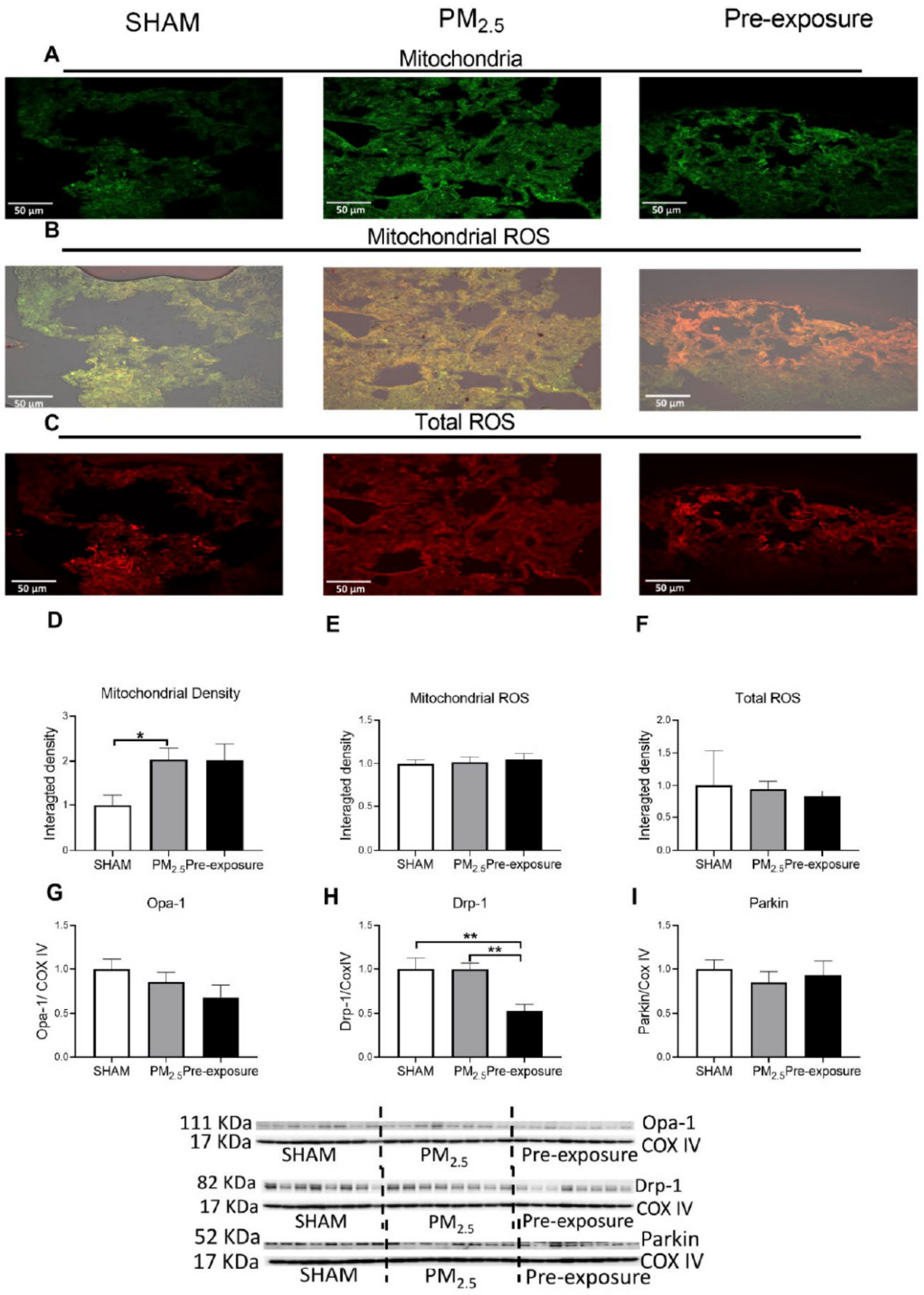

Figure S3. Maternal PM2.5 exposure changed mitochondrial density in the male offspring at 13 weeks. Representative images of (A) mitochondria (green), (B) mitochondrial specific ROS (orange), and (C) total ROS staining (red) and (D-F) quantitative results. Protein levels of (G) Opa-1, (H) Drp-1 and (I) Parkin. Data were analysed by one-way ANOVA followed by Fisher's LSD post hoc tests. *$p < 0.05$, **$p < 0.01$. N=5 in mitochondrial fluorescence staining, N=8 in western blot. Drp-1, dynamin-related protein; Opa-1, optic atrophy-1; $PM_{2.5}$: dams exposed to $PM_{2.5}$ (5μg/day) prior to mating for 6 weeks, during gestation and lactation. Pre-exposure: dams exposed to $PM_{2.5}$ for 6 weeks prior to mating.

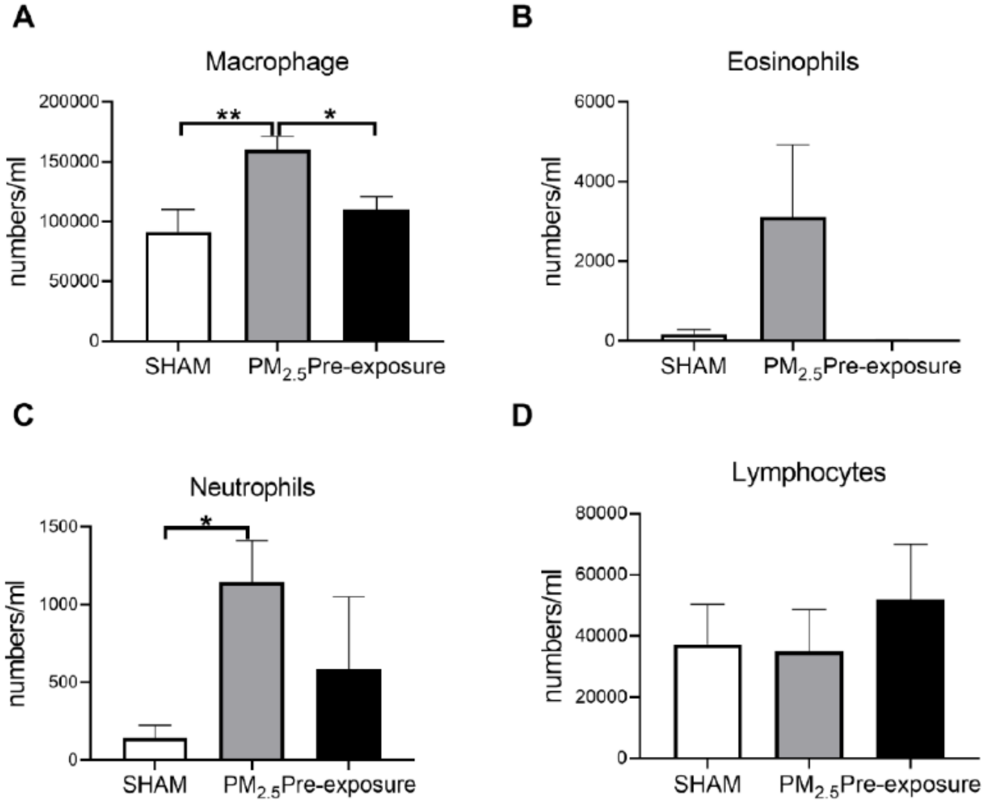

Figure S4. The leukocyte numbers in the male offspring at 13 weeks. The number of (A) macrophages, (B) eosinophils, (C) neutrophils and (D) lymphocytes in the male offspring. Results are expressed as means ± SEM. Data were analysed by one-way ANOVA followed by Fisher's LSD post hoc tests. N=8. *$p < 0.05$, **$p < 0.01$. $PM_{2.5}$: dams exposed to $PM_{2.5}$ (5μg/day) prior to mating for 6 weeks, during gestation and lactation. Pre-exposure: dams exposed to $PM_{2.5}$ for only 6 weeks prior to mating.
